# Peripheral Medulloepithelioma: A Rare Entity to Know

**DOI:** 10.1155/2020/6817407

**Published:** 2020-06-26

**Authors:** W. Matrane, S. Cherkaoui, M. Regragui, N. Bennani Guebessi, M. Karkouri, S. Salam, A. Madani, A. Quessar, N. Khoubila

**Affiliations:** ^1^Hematology and Pediatric Oncology Department, 20th August 1953 Hospital, University Hospital Center Ibn Rochd Casablanca, Morocco; ^2^Pathologic Anatomy and Cytology Laboratory, University Hospital Center Ibn Rochd Casablanca, Morocco; ^3^Pediatric Radiology Department, University Hospital Center Ibn Rochd Casablanca, Morocco

## Abstract

According to the World Health Organization, medulloepithelioma belongs to the embryonal neoplasm entity. It is a very rare, highly malignant tumor typically affecting infants and young children. Usually, the tumor arises in the eye or in the central nervous system; a peripheral location has been rarely reported without an established treatment. The recognition and separation of this neoplasm from other differential tumors are mandatory for better understanding of its biology and determination of optimal treatment. This paper reports a case of an ectopic intrapelvic medulloepithelioma with liver metastasis in a 3-year-old girl.

## 1. Introduction

Medulloepithelioma (ME) is a very rare, highly malignant embryonal tumor of infancy and childhood [1], containing characteristic structures which mimic the appearance of primitive neural tube [2]. Usually, the tumor arises in the ciliary body of the eye or in the central nervous system, although peripheral location has been rarely described [3]. Neoplasm location is an important prognostic factor for survival [4]. The prognosis of central nervous system medulloepithelioma is poor, while intraocular medulloepitheliomas are associated with an excellent prognosis due to the usual possibility of total excision and their natural benign character [5]. Fifteen cases of peripheral medulloepithelioma (PME) have been reported, of which eleven cases were arising from the sacrum and presacral regions. Due to the lack of recognition of these neoplasms, they are often confused with other, less aggressive, neuroectodermal tumors. Therefore, good diagnosis and specific classification are necessary for an optimal treatment of medulloepithelioma. Due to their rarity, reports on medulloepithelioma are most often single cases. We would like to share this unusual case with the medical community for a better recognition and management of this malignant tumor. This paper reports a case of an ectopic intrapelvic medulloepithelioma in a 3-year-old girl.

## 2. Case Report

A 3-year-old female presented to our unit with a four-month history of intermittent abdominal pain accentuated at the time of saddle and urination. Physical examination showed a child with a good general condition. The head, neck, chest, and heart were normal. The abdomen was soft with no mass. There were no palpable lymph nodes and no hepatosplenomegaly. The seat examination showed a hard, presacral mass, palpable in part at the level of the right buttock.

**Figure 1:**
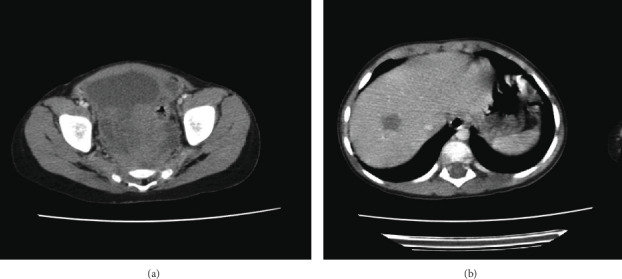
CT scans showing the tumor (a) and the metastatic liver lesion (b) at diagnosis.

Complete blood count, bone marrow aspiration, urinary vanillylmandelic acid (VMA) clearance, blood *β*HCG, alpha foetoprotein, electrolytes, alkaline phosphatase, and liver and kidney functions were all within normal limits. A computed tomography (CT) scan of the chest, abdomen, and pelvis showed a 74^∗^76^∗^155 mm noncalcified presacral soft tissue mass with a 50^∗^43^∗^47 mm metastatic nodule within the liver (Figure 1). The scintigraphy showed a focus of concentration of MIBG in the left paramedian pelvis, with no hyperfixation next to the skeleton or liver.

CT-guided biopsy was performed twice in the presacral mass. The first pathology revealed a round cell tumor proliferation-expressing cytokeratin and CD56. CD45 was negative. This profile is suggestive of a neuroblastoma. However, a round cell desmoplastic tumor cannot be eliminated since Desmin was not tested. The second biopsy showed a malignant tumor proliferation with small round cells expressing CKAE1/AE3, CD56, and CD99. The main differential diagnoses include a tumor of the Ewing family or Ewing-like cell sarcoma. Considering the abdominal location of the tumor, the positivity of the CD56 marker, and the captation on the MIBG scintigraphy, we decided to retain the diagnosis of neuroblastoma and to proceed with first-line chemotherapy according to neuroblastoma protocol (COG A3961) which include 4 chemotherapy cycles, alternating carboplatin/etoposide course and carboplatin/cyclophosphamide/doxorubicin course.

After 4 chemotherapy courses, evaluation scans of the pelvis and abdomen showed a stable aspect of the tumor and of the hepatic metastasis.

**Figure 2:**
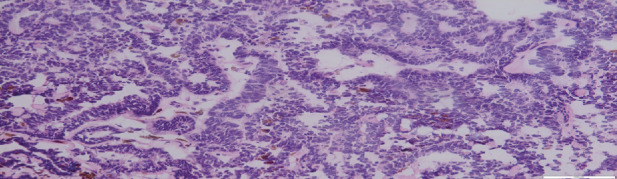
Low magnification: tumor proliferation arraigned in papillary, tubular configuration.

**Figure 3:**
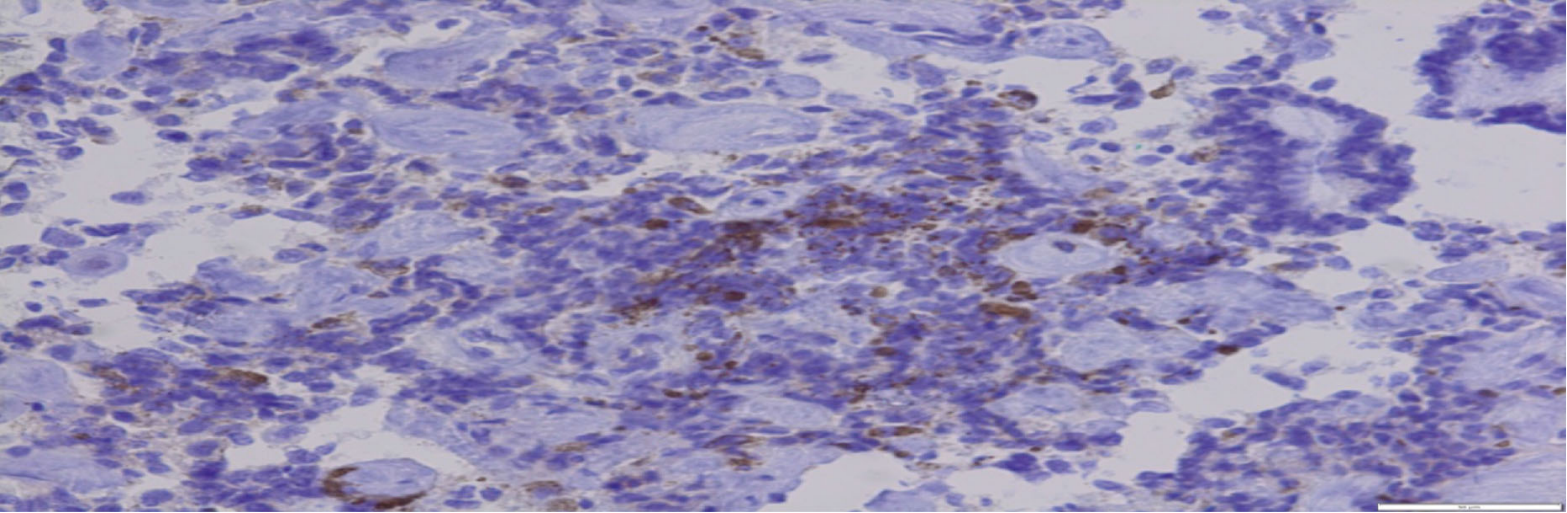
At higher magnification: the cells have a low cytoplasm and hyperchromatic nuclei, conferring a basophilic appearance to the tumor.

**Figure 4:**
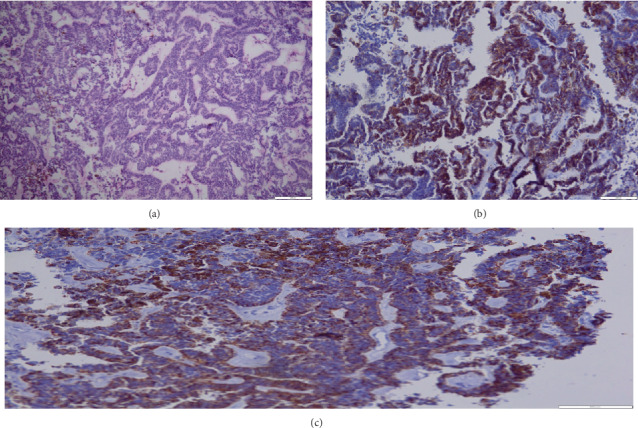
Positivity of neoplastic cells (a) for the cytokeratin AE1AE3 (clone AE1AE3), (b) for CD56 (clone 123C3), and (c) for CD99 (clone 12E7).

A liver biopsy was then performed that histologically consisted of primitive pseudostratified neuroepithelial cells, arranged in papillary and tubular configurations (Figures 2 and 3). Immunohistochemically, the tumor cells were positive for PS100, CKAE1AE3, CD56, SALL4, Glypican-3, SOX-2, cytokeratin AE1AE3, CD56, and CD99 (Figure 4). The synaptophysin, desmine, alpha foetoprotein, PALP, and OCT3/4 were negative. The diagnosis retained in this case was a peripheral medulloepithelioma.

Her treatment regimen included two courses of chemotherapy ICE (ifosfamide, carboplatin, and VP16) followed by a partial resection of the primitive tumor, with an uneventful postoperative course. The pathology confirmed the diagnosis of medulloepithelioma with the same characteristics of the primary tumor.

Immediate radiotherapy and maintenance treatment were scheduled, but unfortunately, the patient's parents decided to give up treatment and go out against medical advice.

## 3. Discussion

Medulloepithelioma was described for the first time as a separate category by Bailey and Cushing in 1926 [6]. According to the World Health Organization, this tumor belongs to the embryonal neoplasm entity [7], mimicing characteristic structures of an embryonic neural tube [8]. Such neoplasm appears only in sites where an undifferentiated medullary epithelium persists [9]. It is a rare and highly malignant tumor, with a rapid growth and tendency to invade the meninges. This tumor affects young children between 6 months and 5 years of age [10]. Incidence is equal in males and females [9]. The most common localizations reported for medulloepithelioma are to the periventricular regions of the brain and ciliary body of the eye [1, 2]. So far, only a few reports of peripheral medulloepithelioma have been described in literature, with sites including the presacral region and pelvis [3–8] (Table 1). Histologically, medulloepitheliomas are characterized by their resemblance to the embryonic neural tube, arranged in papillary, tubular, or trabecular configuration with an internal and external limiting membrane [10]. The presence of the external membrane, highlighted with periodic acid Schiff-positive internal limiting membrane, is very helpful for the diagnosis of a medulloepithelioma and for differential diagnosis with other neuroectodermal tumor derivation, such as primitive neuroectodermal tumors [2]. Medulloepithelioma cells express vimentin, nestin, and microtubule-associated protein 5; some tumors also express neurofilaments but not S-100 protein, epithelial markers, or glial fibrillary acidic protein [10–12]. This neoplasm should be differentiated from primitive neuroectodermal and germinal cell tumors.

In the 2016 WHO classification revision of CNS tumors, substantial changes have been introduced in the classification of many categories including embryonal tumors other than medulloblastomas, based on molecular parameters and histology [13].

Primitive CNS tumors including embryonal tumor with multilayered rosettes (ETMR) and medulloepithelioma share the same molecular and genetic characteristics including the diffuse expression of LIN28A, amplification of the C19MC region on chromosome 19 (19q13.42), and DNA-methylation profiles most often showing numerous copy number aberrations which affect most commonly chromosomes 19 and 2 [14]. However based on their location, ME and ETMP may be divergent immunohistochemically and genetically. LIN28A has been shown to be a pertinent biologically marker for both ME and ETMRs despite the cellular origin, while the amplification of the 19q13 locus is specifically a characteristic of ETMR C19MC, altered and exhibited by only some cases of ME [4].

In the absence of C19MC alteration, a neoplasm with histological features conforming to the embryonal tumor with multilayered rosettes should be recognized as ETMR-NOS, and a tumor with histological features of medulloepithelioma should be diagnosed as ME [15].

Fifteen cases of PME have been reported. Surgery was performed in thirteen cases. Chemotherapy and radiotherapy was received, respectively, by twelve and five patients. Autograft was performed in one case which have relapsed 6 months later.

Five patients died of the disease; nine were disease free with a median follow-up of 50 months, while one patient was still on treatment.

Patients were treated with a diverse range of therapeutic agents and different chemotherapy drugs resulting in several outcomes, making it difficult to infer the efficiency of each therapeutic option.

Regarding our patient, the case was challenging in both diagnostical and treatment levels. In our context, we were unable to perform gene amplification studies.

After redressing the diagnosis, the child had partial resection of the tumor followed by adjuvant chemotherapy (in pre- and postoperative). Unfortunately, the tumor was chemoresistant and the patient's parents decided not to pursue any other treatment options.

Due to the rarity of this neoplasm, optimal management is yet to be established. The expression of platelet-derived growth factor receptor (PDGFR) and other tumor target proteins was reported, suggesting a potential targeting treatment with tyrosine kinase inhibitor [3] and mTOR inhibitors in the context of LIN28 signaling [4]. Surgical excision is the standard care, and a complete surgical removal remains the aim of surgery. Adjuvant radiation or chemotherapy or a combination is often advocated to optimize tumor control and survival. Prognostic data are limited, but the central nervous system medulloepitheliomas are known to be associated with a good prognosis only if completely resected. The prognosis of PME is intermediate between the ocular forms (of benign evolution) and central nervous system medulloepitheliomas (aggressive).

## 4. Conclusion

The diagnosis and the management of such neoplasms are very challenging. Presacral medulloepitheliomas are often misclassified and confused with other embryonal tumors. However, with the widespread availability of genetic and molecular markers, more cases will be diagnosed.

## Figures and Tables

**Table 1:** Review of literature for peripheral medulloepithelioma cases [3–15].

Reference	Age	Gender	Site	Stage	Metastasis	Therapy	Outcome
Kleinman et al. [11]	20 yr	F	Ovary	I	No	Complete surgery, CT	NED, 108 mo
Kleinman et al.	32 yr	F	Ovary	I	No	Complete surgery, CT	NED, 36 mo
Kleinman et al.	13 yr	F	Ovary	III	No	Complete surgery	DOD, 20 mo
Kleinman et al.	23 yr	F	Ovary	III	No	Complete surgery, CT, RT	DOD, 2 mo
Figarella-Branger et al. [16]	17 yr	F	Pelvis	IV	Lung	Surgery on primitive tumor	DOD, 8 mo
Bruggers et al. [5]	Birth	F	Pelvis	III	No	Partial resection, CT	NED, 50 mo
Donner and Teshima [12]	12 yr	F	Pelvis	III	No	CT	NED, 36 mo
Nakamura et al. [8]	6 mo	M	Pelvis (sciatic nerve)	III	No	Complete surgery	NED, 84 mo
De Pasquale et al. [3]	3 yr	F	Retroperitoneal mass	III	No	Surgery, CT, autograft, RT	NED, 6 mo, then replase
Pillai et al. [10]	3 yr	F	Pelvis	I	No	Complete surgery, CT, RT	NED, 5 mo
Seemayer et al. [7]	5 yr	F	Presacral	—	No	Surgery, RT, CT (after metastasis)	Liver metastases after 2 mo, died 8 mo after diagnosis
Somjee et al. [17]	3 yr	M	Presacral	IV	Liver and lung metastases	CT	On treatment (8 mo)
Tran et al. [18]	5 yr	F	Sacrococcygeal	—	No	Surgery, RT	Recurrence 6 yr after diagnosis, liver metastasis
Lee et al. [19]	Birth	M	Sacrococcygeal	—	—	Surgery, CT	DOD, cerebral metastasis
Honnart et al. [4]	2 yr	F	Presacral	—	No	CT, surgery, rapamycin maintenance	NED 5 yr
Our case	3 yr	F	Presacral	IV	Liver	Partial surgery, CT	Alive

Legend: F: female; M: male; yr: years; mo: months; CT: chemotherapy; RT: radiotherapy; NED: nonevidence of disease; DOD: died of disease.
